# Sociocultural Influences on Moral Judgments: East–West, Male–Female, and Young–Old

**DOI:** 10.3389/fpsyg.2016.01334

**Published:** 2016-09-05

**Authors:** Karina R. Arutyunova, Yuri I. Alexandrov, Marc D. Hauser

**Affiliations:** ^1^Laboratory of Neural Bases of Mind, Institute of Psychology – Russian Academy of SciencesMoscow, Russia; ^2^Department of Psychology, National Research University Higher School of EconomicsMoscow, Russia; ^3^Risk-Eraser, LLC, West FalmouthMA, USA

**Keywords:** morality, moral judgment, cross-cultural, individual differences, gender, age

## Abstract

Gender, age, and culturally specific beliefs are often considered relevant to observed variation in social interactions. At present, however, the scientific literature is mixed with respect to the significance of these factors in guiding moral judgments. In this study, we explore the role of each of these factors in moral judgment by presenting the results of a web-based study of Eastern (i.e., Russia) and Western (i.e., USA, UK, Canada) subjects, male and female, and young and old. Participants (*n* = 659) responded to hypothetical moral scenarios describing situations where sacrificing one life resulted in saving five others. Though men and women from both types of cultures judged (1) harms caused by action as less permissible than harms caused by omission, (2) means-based harms as less permissible than side-effects, and (3) harms caused by contact as less permissible than by non-contact, men in both cultures delivered more utilitarian judgments (save the five, sacrifice one) than women. Moreover, men from Western cultures were more utilitarian than Russian men, with no differences observed for women. In both cultures, older participants delivered less utilitarian judgments than younger participants. These results suggest that certain core principles may mediate moral judgments across different societies, implying some degree of universality, while also allowing a limited range of variation due to sociocultural factors.

## Introduction

Social norms, both implicit and explicit, guide individual behavior. From early stages of development and throughout life we learn to adapt our behavior according to social expectations and requirements, which may differ across cultures and may be relevant in various degrees for men and women of different ages. On the other hand, social and moral norms are part of every human culture, with some indication that a set of core principles may underlie fundamental moral judgments. On this view, some aspects of moral competence may be universal, part of the human endowment (e.g., Dwyer, 1999; Hauser, 2006; Mikhail, 2011). In this study, we explore whether cultural beliefs, gender and age impact moral judgments, and if so, how.

Morally relevant judgments and actions appear to be based on different cognitive processes when compared with other rule-based social interactions such as conventional situations (Turiel, 1983; Huebner et al., 2010; FeldmanHall et al., 2012; Young and Dungan, 2012; Pascual et al., 2013). While conventional rules may vary significantly across cultures and social groups, some moral principles are hypothesized to be unconsciously operative and, to a significant extent, universal. On this view, these principles are part of our core endowment as a species, what some have referred to as our universal moral grammar (Rawls, 1971; Dwyer, 1999; Harman, 1999; Mikhail, 2000, 2007, 2011; Hauser, 2006, etc.) The strong version of this thesis is that such principles are largely immune to sociocultural influences. An alternative view is that both moral judgments and actions are culturally constructed through conscious and rational deliberation (Kohlberg, 1981, 1984). On this view, what is universal is limited to some sense of right and wrong and some way of encouraging or supporting morally appropriate actions while discouraging or punishing morally inappropriate actions. It is up to each culture to decide what is morally appropriate.

Understanding the nature and development of our moral sense is complicated not only by the different theoretical perspectives noted above, but because of different methodologies. For example, some studies focus on moral judgments and others on moral actions; some present hypothetical moral situations and others real-world moral cases; and some test a narrow range of subjects, mostly from Western developed nations, while others sample small-scale societies. Our goal in this paper is to focus on moral judgments, using a well-studied battery of hypothetical moral dilemmas, while extending the analysis to an Eastern culture, along with potential age and gender effects. We begin by discussing some of the relevant literature, focusing especially on the inconsistency of results with respect to the contribution of gender, age, and cultural beliefs in guiding moral judgments.

### The Role of Gender

Gilligan (1977, 1982) initiated the debate about gender differences in moral judgments in response to Kohlberg’s (1969) original work on the problem. She suggested that because of differences in early socialization, boys develop into independent agents whose behavior is regulated by rights and duties. In contrast, girls are more focused on their network of social relationships, placing a greater emphasis on social responsibility and care as opposed to justice. Although, Gilligan’s position found little empirical support (Jaffee and Hyde, 2000), gender differences in moral behavior can be found in other literatures. For example, studies demonstrate that young boys lie more often than young girls (Gervais et al., 2000). In adolescence, boys violate moral rules, including harming other people, more often than girls (Moffitt et al., 2001). Adult men are involved in significantly more crime, associated with severe moral violations, than women (e.g., Bennett et al., 2005). As for moral judgments, however, results are mixed. In some studies, men are less likely than women to support altruistic actions in every-day scenarios (Rosen et al., 2016) and are more likely to deliver utilitarian responses to hypothetical, harm-based moral scenarios (Fumagalli et al., 2010; Youssef et al., 2012; Friesdorf et al., 2015). In contrast, other studies using similar scenarios and methodologies show that gender, as well as several other cultural factors (age, political affiliation, religious background), contribute very little to the pattern of judgments observed (Hauser et al., 2007; Banerjee et al., 2010; Gleichgerrcht and Young, 2013). Lastly, fMRI data suggest that even when delivering similar behavioral responses, there are sex differences in neural processing (Harenski et al., 2008); this result suggests that judgment data may hide underlying gender differences, at least in some cases. In sum, though many studies consistently show gender differences in moral action, the role of gender in the formation of moral judgments is much less clear.

### The Role of Age

It has long been established that from an early age, children can differentiate moral violations and conventional transgressions (e.g., Smetana, 1983; Smetana and Braeges, 1990; Gasser and Keller, 2009), and distinguish the same actions resulting in different outcomes (e.g., Nelson, 1980) as well as actions with the same outcome but different intentions (Armsby, 1971; Cushman et al., 2013). What is less clear is whether and how patterns of moral judgment change throughout life, in part because of the variety of methods deployed.

In studies using hypothetical moral dilemmas involving harm to others, most results show little to no change across development. In a study of largely Western subjects, varying in age from 10 to 87, individuals judged (1) harm caused by action as worse than equivalent harm caused by omission (action/omission); (2) harm intended as a means as worse than harm foreseen as a side effect (means/side effect); and (3) harm caused via physical contact as worse than harm caused without contact (contact/non-contact; Cushman et al., 2006; Hauser et al., 2009). Though there were no significant age effects among this sample for these three distinctions or principles, the majority of subjects were adults. In another study focusing on different variants of the trolley problem, Hauser et al. (2007) found that age predicted only 1.4% of the variance in moral judgments; here as well, most subjects were adults. Pellizzoni et al. (2009, 2010), showed that by the age of 4–5 years old, children judge means-based harms as worse than side effects, and contact as worse than non-contact. Adolescents (14–18 year olds) apply all three principles in their moral judgments like adults, but vary in their moral justifications (Stey et al., 2013). These data support the idea that for hypothetical moral dilemmas involving harm, intuitive moral judgments appear early in development, and as a cognitive process, emerge prior to deliberative, conscious justification, which develops later along with other cognitive abilities, including language skills.

Using different moral dilemmas and methodologies of presentation, Pratt et al. (1987) found no age difference in stage level of moral development and reported role-taking on hypothetical moral dilemmas, but older individuals (60–75 years old) reported more varied reflections on their personal experience at solving moral dilemmas in real life. Chap (1986) reported a significant difference between elderly and early middle-aged individuals on a measure of spontaneous role taking when assessing moral dilemmas, with the elderly making more definitive moral judgments than younger adults, who tended to reconcile various points of view represented in dilemmas. Narvaez et al. (2011) showed that older individuals (60–82 years old) demonstrate an enhanced memory for morally charged events as compared to non-moral events; they are also more likely to apply moral background knowledge to understanding presented events. Finally, in their recent study Rosen et al. (2016) found that age significantly correlated with more altruistic moral decisions.

Additional evidence of age differences in moral judgments has emerged from neuroimaging studies. Harenski et al. (2012) investigated the dynamics of brain activity during the evaluation of images involving moral and non-moral transgressions. Results showed that the patterns of activation varied across participants of different age, from 13 to 53. Specifically, there was a significant positive correlation between age and hemodynamic activity in brain areas associated with mentalizing as well as emotional and self-reflective processing. In addition, Decety et al. (2012) showed that brain areas engaged in moral judgment become more functionally coupled with age.

In sum, it is presently unclear whether and how moral judgments may change during development, including especially development in adolescence through adulthood. The primary reason for this ambiguity is the diversity of approaches involved in assessing this problem. Due in part to the consistency of results for studies involving hypothetical, harm-based moral dilemmas, and especially the lack of age effects, this study further explores the role of age using the same methodology.

### The Role of Cultural Beliefs and Norms

Social and even simple perceptual judgments vary cross-culturally, including especially contrasts between East and West (e.g., Nisbett et al., 2001; Kitayama and Uskul, 2011). The key dimension relevant to such cross-cultural comparisons is individualism versus collectivism (Triandis, 1995), or independence versus interdependence (Markus and Kitayama, 1991). Western cultures prioritize independence and individualistic values, such as self-promotion, self-expression, and self-sustenance (e.g., Kitayama and Uskul, 2011). Eastern cultures, in contrast, emphasize interdependence between individuals and collectivistic values, such as social harmony, duties, and relational attachment. Russian culture has distinctive features that are typical of Eastern collectivism (Tower et al., 1997; Matsumoto et al., 1998; Alexandrov and Alexandrova, 2009; Varnum et al., 2009; Grossmann and Varnum, 2011) and may be of particular interest for cross-cultural comparisons of moral judgments due to the fact that it is a large scale, developed society, comparable to Western societies. At the same time it is necessary to emphasize that each culture within the Western and Eastern types has its distinctive features which may vary significantly, therefore data obtained in the Russian population cannot be directly generalized to other Eastern cultures, such as China or Japan. However, such data may be useful in considering common tendencies typical for collectivistic societies. For example, in other studies Chinese participants are less utilitarian than Western participants (e.g., Ahlenius and Tännsjö, 2012; Gold et al., 2014). In case of the classical trolley problem moral judgments varied in the USA (killing one to save five was judged as permissible by 81% of respondents), Russia (63%) and China (52%), suggesting that in general Eastern cultures as compared to Western may be less utilitarian but in various degrees.

In cross-cultural analyses, it is important to isolate which particular factors lead to what is called “cultural differences.” For example, in a series of studies using subjects responding to morally salient scenarios presented on the internet, there was virtually no impact of gender, age, religious background, or political affiliation on moral judgments of hypothetical harm-based scenarios (Hauser et al., 2007; Banerjee et al., 2010). In particular, these cultural variables had no impact on the three morally central distinctions mentioned above (means-side effects, action-omission, and contact-noncontact). However, the majority of subjects were from Western, English-speaking countries, including the USA, Canada, UK, and Holland (Cushman et al., 2006; Hauser, 2006; Hauser et al., 2009). As noted above, individuals from a small-scale rural Mayan population judged means-based harms as worse than harms resulting from side-effects — paralleling both Western subjects as well as a more educated urban Mayan population — but judged action-based harms as comparable to omissions (Abarbanell and Hauser, 2010). This difference in judgments for the action-omission cases could be due to something particular about Mayan culture or reflect differences that characterize all small-scale societies, demonstrating that looking into different cultures may provide insights into the nature of moral judgments. For example, in a recent study of several small scale societies (ranging from hunter-gatherers to horticulturalists), results revealed considerable variation in the pattern of moral judgments concerning scenarios emphasizing the role of intention (Barrett et al., 2016). Based on these results, the authors conclude that our current, modern emphasis on intentionality may reflect a culturally evolved process as opposed to a system that is part of our innate endowment. Given the different results and methodologies, it is difficult to discern at present how, and to what extent, cultural factors impact moral judgments and actions.

This paper presents an in-depth analysis of data on moral judgments collected in two web-based studies, one of Western English-speaking countries (Cushman et al., 2006) and the other of a Russian sample (Arutyunova et al., 2013). Our goal was to examine whether gender, age and the East–West axis have a significant impact on the nature of moral judgments and if so, in what way. A strength of this analysis is that because all subjects were tested using the same methodology (i.e., web-based presentation, same controlled hypothetical moral dilemmas involving harm, same judgment scales), any differences are more likely to be due to the sociocultural factors examined.

## Materials and Methods

### Participants

Men and women of different age, religious, educational, occupational, and other demographic groups voluntarily participated in an Internet-based study (Cushman et al., 2006; Arutyunova et al., 2013). Of these subjects, we analyzed the data from 659 subjects who fully completed a demographic questionnaire, provided judgments of permissibility for all moral dilemmas, and correctly answered two control scenarios intended to test attention and understanding of instructions.

The Russian sample included 89 male (16–69 years old, *M* = 28, *SD* = 12) and 238 female (16–58 years old, *M* = 27, *SD* = 10) participants. The Western English-speaking sample included 191 male (10–85 years old, *M* = 37, *SD* = 15) and 141 female (14–66 years old, *M* = 38, *SD* = 14) participants. For the analysis we divided the two samples into five age groups (**Table 1**): 10–19, 20–24, 25–34, 35–44, and 45–85 years old.

**Table 1:** Five age groups.

Age group	Years old	Description	*N*		Female bias, %		Age, *M (SD*)		Age, Med	
			Rus	West	Rus	West	Rus	West	Rus	West
1	10–19	Adolescents and teenagers	93	38	72	37	17.6 (1.0)	16.9 (2.3)	18	17
2	20–24	Young adults	86	32	76	53	21.8 (1.3)	22.0 (1.6)	22	22.5
3	25–34	Adults	81	85	73	40	29.3 (3.0)	29.3 (2.8)	29	29
4	35–44	Middle age adults	40	68	80	35	38.9 (3.0)	39.4 (2.7)	39	39
5	45–85	Older adults	27	109	56	48	52.9 (6.1)	54.8 (7.2)	51	54

*Rus, Russian sample; West, Western sample.*

### Experimental Procedure

Participants voluntarily logged onto the web-site (moral.wjh.harvard.edu, for more details about experimental design and procedures, see Cushman et al., 2006) where they followed on-screen instructions to fill in a demographic questionnaire with information on gender, age, religion, education and political affiliation. They were next presented with 32 moral scenarios (for content of original scenarios in English and their translation into Russian, see Appendix in Arutyunova et al., 2013) in a randomized order. Of these scenarios, 30 involved a situation where a protagonist made a choice to sacrifice one person in order to save five other people. These scenarios comprised 18 controlled pairs differentiating (1) actions versus omissions, (2) intended means versus foreseen side-effects, and (3) contact versus no contact (see Cushman et al., 2006; Hauser et al., 2009). The two remaining scenarios served as controls, presented non-moral situations, and provided one mechanism to assess whether participants understood the instructions and were paying attention. An example of one of the 30 scenarios is as follows:

“Luke is operating the switch at a railroad station when he sees an empty, out of control boxcar coming down the tracks. It is moving so fast that anyone it hits will die immediately. The boxcar is headed toward five repairmen on the track. If Luke does nothing, the boxcar will hit the five repairmen on the track. Luke can pull a lever redirecting the boxcar to an empty sidetrack. However, pulling the lever will cause the switch to crush one other repairman working on the switch, who will die immediately. Luke decides to pull the lever.

Pulling the lever is: 1 (Forbidden) – 2 – 3 – 4 (Permissible) – 5 – 6 – 7 (Obligatory)”

Participants were instructed to read the scenarios and then decide, using a seven-point Likert scale, how they would rate the protagonist’s behavior. The scale was anchored on one end by “forbidden” and at the opposite end by “obligatory,” with “permissible” anchoring the mid-point of the scale.

### Data Analysis

We analyzed moral judgments in relation to gender, age, and culture (Russian versus Western). The analysis included comparisons of the pairs of scenarios, moral permissibility ratings (MPRs) and extreme judgments.

Eighteen controlled pairs of scenarios differentiating (1) actions and omissions, (2) intended means and foreseen side-effects, and (3) contact and no contact were compared with paired-sample *t*-test, within subjects (see Cushman et al., 2006).

The MPRs (see Paxton et al., 2012 for similar analysis) were calculated as a mean score across all 30 test scenarios within each subject^1^; and were used to describe overall moral judgments about all different types of harms included in this study (i.e., those caused by action or omission, intended as means or foreseen as a side effect, via physical contact or no contact). We intended to see how the permissibility of utilitarian actions and omissions in general was assessed within our groups. Prior to analyzing the MPRs we calculated Chronbach’s alpha to ensure significant reliability of responses across 30 scenarios in both, Russian and Western samples.

Extreme judgments – the scores on the borders of the seven-point scale – included utilitarian extreme (7 = “Obligatory”) and non-utilitarian, or deontological, extreme (1 = “Forbidden”) and were used as an additional variable to complement the MPR analysis and show how the factors of gender, age, and culture are reflected in response style when evaluating moral dilemmas. We analyzed the number and percentages of these two types of extreme moral judgments in different groups of participants.

We used IBM SPSS.20 for statistical analyses. Distributions were tested for normality with Kolmagorov–Smirnov test. Chronbach’s alpha was used to assess reliability. A univariate general linear model (a three-way ANOVA) was used to determine the role of the factors of gender, age, and culture. Two groups were compared with independent *t*-test, or alternatively with non-parametric Mann–Whitney tests. For three or more groups, a one-way ANOVA was performed followed by *post hoc* Bonferroni tests, or alternatively we used non-parametric Kruskal–Wallis test followed by paired Mann–Whitney comparisons. Leven’s test was used to check for homogeneity of variance, and Welch statistic was calculated additionally for groups with heterogeneous variances. In-group comparisons were done with Wilcoxon tests. Jonckheere trend test was used to study the dynamics of MPRs in different age groups. Pearson’s correlation was used to establish association between variables. The following estimates of effect size were calculated: Cohen’s d with *t*-test; ω with ANOVA; association coefficient (r) with non-parametric tests. Significance level at *p* < 0.05.

## Results

First, we calculated Chronbach’s alpha for all 30 test scenarios and showed that responses to these scenarios had strong reliability in both Russian (Chronbach’s α = 0.93) and Western samples (Chronbach’s α = 0.96). We next averaged all 30 responses given by each subject into one single MPR value. Thus, MPRs were calculated for each subject, and we used these values as general indicators of moral permissibility of various harmful behaviors toward one person resulting in a greater good of saving five other people.

To analyze variance in MPRs in relation to the factors of culture, gender and age group we used a univariate general linear model (a three-way ANOVA). Results showed significant main effects of all three factors (**Table 2**), culture [*F*_1_(1,658) = 24.023, *p* < 0.001, ω = 0.048], gender [*F*_2_(1,658) = 16.218, *p* < 0.001, ω = 0.039] and age group [*F*_3_(4,658) = 6.075, *p* < 0.001, ω = 0.045]. The interaction of the factors was not significant (see **Table 2**). As the assumption of homogeneity of variance was not met [Leven’s test, *F*(3,655) = 8.04, *p* < 0.001], we additionally performed a Welch ANOVA separately for each independent variable and also received significant effects of culture [Welch statistics (1,642.451) = 23.465, *p* < 0.001] and gender [Welch statistics (1,511.885) = 32.881, *p* < 0.001]. Due to significant cultural differences, the effect of age group was tested separately for Russian and Western samples and results are reported below (see Age Comparisons).

**Table 2:** ANOVA results for the factors of culture, gender, and age group.

Source	Type III sum of squares	df	Mean square	*F*	Significance
Corrected model	100.960	19	5.314	4.591	0.000
Intercept	7208.112	1	7208.112	6227.40	0.000
**Culture**	**27.806**	**1**	**27.806**	**24.023**	**0.000**
**Gender**	**18.772**	**1**	**18.772**	**16.218**	**0.000**
**Age group**	**28.129**	**4**	**7.032**	**6.075**	**0.000**
Culture ^∗^ Gender	2.494	1	2.494	2.154	0.143
Culture ^∗^ Age Group	4.443	4	1.111	0.960	0.429
Gender ^∗^ Age Group	3.940	4	0.985	0.851	0.493
Culture ^∗^ Gender ^∗^ Age group	9.386	4	2.347	2.027	0.089
Error	739.632	639	1.157		
Total	11384.112	659			
Corrected total	840.592	658			

*The bold values indicate those results that are statistically significant, at least at *p* < 0.05; all other *p*-values are not statistically significant.*

### Three Moral Distinctions

Results on the three moral distinctions within the Western and Russian samples were previously reported (see Cushman et al., 2006; Arutyunova et al., 2013 correspondingly). Here, however, we analyzed these data separately for the two genders. Using within subjects *t*-test, we compared how men and women from Russian and Western cultures judged harmful actions and omissions within pairs of moral scenarios. As shown in **Tables 3** and **4**, male and female participants, from both cultures, tended to judge as less permissible, (1) actions as opposed to omissions, (2) means as opposed to side-effects, and (3) physical contact as opposed to without contact. Such analyses were not performed separately within different age groups because some of these groups had an insufficient sample size.

**Table 3:** Differences in permissibility for pairs of moral scenarios in the Russian sample.

	Male					Female				
Scenario pair	Mean difference	SD	*t*(88)	Effect size *(d)*	*p* (two-tailed)	Mean difference	SD	*t*(237)	Effect size *(d)*	*p* (two-tailed)
**Inaction – action**					
Boxcar	0.92	2.01	4.33	0.46	**0.000**	1.02	1.69	9.31	0.60	**0.000**
Pond	1.43	1.78	7.55	0.80	**0.000**	1.51	1.85	12.61	0.82	**0.000**
Ship	0.33	3.01	1.02	0.11	0.310	0.03	2.75	0.17	0.01	0.869
Car	0.66	1.59	3.94	0.42	**0.000**	0.81	1.75	7.17	0.46	**0.000**
Boat	0.48	2.15	2.12	0.22	**0.037**	0.30	2.01	2.29	0.15	**0.023**
Switch	0.16	1.74	0.85	0.09	0.395	0.13	1.89	1.10	0.07	0.273
**Side effect -means**					
Speedboat	0.35	1.58	2.08	0.22	**0.041**	0.59	1.40	6.47	0.42	**0.000**
Burning	1.24	1.69	6.89	0.73	**0.000**	1.34	1.83	11.31	0.73	**0.000**
Boxcar	0.83	1.96	4.01	0.43	**0.000**	0.82	1.60	7.93	0.51	**0.000**
Switch	0.28	1.60	1.65	0.18	0.102	0.25	1.50	2.55	0.17	**0.011**
Chemical	0.48	1.89	2.41	0.26	**0.018**	0.21	1.51	2.10	0.14	**0.037**
Shark	0.49	1.54	3.03	0.32	**0.003**	0.50	1.51	5.14	0.33	**0.000**
**No contact -contact**					
Speedboat	0.99	1.55	6.02	0.64	**0.000**	0.79	1.47	8.26	0.54	**0.000**
Intended burning	0.56	1.63	3.25	0.34	**0.002**	0.41	1.61	3.90	0.25	**0.000**
Boxcar	0.80	1.44	5.23	0.55	**0.000**	0.76	1.40	8.42	0.55	**0.000**
Foreseen burning	0.79	1.74	4.28	0.45	**0.000**	0.50	1.56	4.94	0.32	**0.000**
Aquarium	0.12	1.47	0.79	0.08	0.429	0.22	1.30	2.59	0.17	**0.010**
Rubble	0.42	1.66	2.37	0.25	**0.020**	0.12	1.26	1.44	0.09	0.150

*Significant differences are highlighted in a darker shade of gray. The bold values indicate those results that are statistically significant, at least at *p* < 0.05; all other *p*-values are not statistically significant.*

**Table 4:** Differences in permissibility for pairs of moral scenarios in the Western sample.

	Male					Female				
Scenario pair	Mean difference	*SD*	*t*(190)	Effect size *(d)*	*p* (two-tailed)	Mean difference	*SD*	*t(140)*	Effect size *(d)*	*p* (two-tailed)
**Inaction – action**				
Boxcar	0.80	2.19	5.02	0.36	**0.000**	0.58	1.79	3.85	0.32	**0.000**
Pond	1.80	2.10	11.88	0.86	**0.000**	1.53	1.87	9.73	0.82	**0.000**
Ship	0.66	2.19	4.17	0.30	**0.000**	1.06	1.71	7.40	0.62	**0.000**
Car	1.01	1.79	7.79	0.56	**0.000**	0.75	1.74	5.13	0.43	**0.000**
Boat	1.05	1.94	7.48	0.54	**0.000**	0.87	2.03	5.09	0.43	**0.000**
Switch	0.35	1.98	2.45	0.18	**0.015**	0.14	1.71	0.99	0.08	0.326
**Side effect -means**				
Speedboat	0.24	1.20	2.77	0.20	**0.006**	0.36	1.06	4.04	0.34	**0.000**
Burning	1.20	1.69	9.84	0.71	**0.000**	1.30	1.69	9.10	0.77	**0.000**
Boxcar	0.59	1.80	4.54	0.33	**0.000**	0.37	1.50	2.91	0.25	**0.004**
Switch	0.34	1.87	2.51	0.18	**0.013**	0.21	1.61	1.51	0.13	0.133
Chemical	0.25	1.58	2.15	0.16	**0.032**	0.23	1.41	1.97	0.17	0.051
Shark	0.16	1.73	1.30	0.09	0.195	0.50	1.81	3.25	0.27	**0.001**
**No contact -contact**				
Speedboat	0.83	1.46	7.81	0.56	**0.000**	0.99	1.39	8.40	0.71	**0.000**
Intended burning	0.24	1.40	2.38	0.17	**0.018**	0.25	1.40	2.11	0.18	**0.037**
Boxcar	1.12	1.78	8.68	0.63	**0.000**	0.99	1.64	7.18	0.61	**0.000**
Foreseen burning	0.34	1.32	3.55	0.26	**0.000**	0.40	1.06	4.52	0.38	**0.000**
Aquarium	0.15	1.52	1.33	0.10	0.185	0.21	1.09	2.25	0.19	**0.026**
Rubble	0.09	1.25	0.99	0.07	0.325	0.12	1.31	1.09	0.09	0.277

*Significant differences are highlighted in a darker shade of gray. The bold values indicate those results that are statistically significant, at least at *p* < 0.05; all other *p*-values are not statistically significant.*

### Gender Comparisons

**FIGURE 1:**
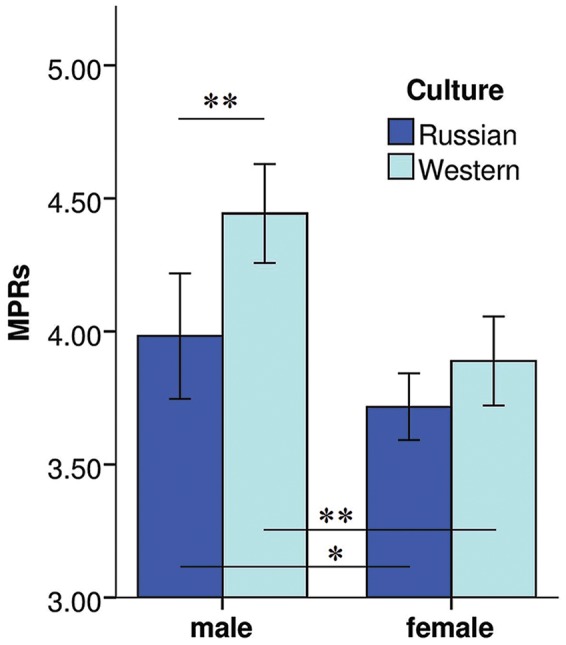
**Gender effects on moral judgments.** Mean MPRs (±2 SE) are shown for men and women in Russian (dark blue rows) and Western (light blue rows) cultures. Men in both cultures delivered more utilitarian judgments. Western men were more utilitarian than Russian men with no cultural difference between women, *t*-test for independent samples, ^∗^*p* < 0.05, ^∗∗^*p* < 0.01.

Overall, men delivered more utilitarian moral judgments (harm one to save five) than women (**Figure 1**), in both Russian [*t*(325) = 2.121, *p* = 0.036, *d* = 0.24] and Western [*t*(329.271) = 4.435, *p* < 0.001, *d* = 0.49] cultures.

We next explored extreme judgments, those on the borders of the scale (1 = “forbidden,” 7 = “obligatory”). Among Russians, both men (Wilcoxon match pairs test, *n* = 89, *T* = 766.5, *Z* = 2.79, *p* = 0.005, *r* = 0.21) and women (Wilcoxon match pairs test, *n* = 238, *T* = 3896, *Z* = 6.53, *p* < 0.001, *r* = 0.30), were more likely to select the “forbidden” end of the scale as opposed to “obligatory.” Similarly, Western women were also more likely to select “forbidden” than “obligatory” (Wilcoxon match pairs test, *n* = 141, *T* = 1576.5, *Z* = 2.88, *p* = 0.004, *r* = 0.17); Western men showed no such difference (Wilcoxon match pairs test, *n* = 191, *T* = 4568.5, *Z* = 1.43, *p* = 0.15).

### Age Comparisons

Age was negatively correlated with MPRs in the Russian sample (Pearson *r* = -0.23, *p* < 0.001; Spearman *r* = -0.28, *p* < 0.001), suggesting that moral judgments become less utilitarian with age. However, no significant correlation was found in the Western sample (Pearson *r* = -0.04, *p* = 0.464; Spearman *r* = -0.11, *p* = 0.055).

To analyze variances in MPRs in relation to age, we used a univariate general linear model (a one-way ANOVA). Results showed that the main effect of age was significant in the Russian culture [*F*(4,322) = 5.360, *p* < 0.001, ω = 0.058], but not in the Western culture [*F*_3_(4,327) = 1.301, *p* = 0.27]. *Post hoc* Bonferroni tests (see **Table 5**) revealed that in the Russian culture, responses of the youngest age group (16*–*19 years old) were different from responses of the older age groups (25–69 years old), but with no significant differences among the older age groups.

**Table 5:** Multiple comparisons of age groups in Russian culture (Bonferroni tests).

Age groups		Mean difference	Standard error	*p*	95% confidence interval	
					Lower bound	Upper bound
16–19	20–24	0.27706	0.14790	0.619	-0.1410	0.6951
	25–34	0.51668^∗^	0.15025	0.007	0.0920	0.9414
	35–44	0.55998^∗^	0.18693	0.030	0.0316	1.0883
	45–69	0.78767^∗^	0.21612	0.003	0.1768	1.3985
20–24	16–19	-0.27706	0.14790	0.619	-0.6951	0.1410
	25–34	0.23962	0.15307	1.000	-0.1930	0.6723
	35–44	0.28291	0.18921	1.000	-0.2519	0.8177
	45–69	0.51061	0.21809	0.198	-0.1058	1.1271
25–34	16–19	-0.51668^∗^	0.15025	0.007	-0.9414	-0.0920
	20–24	-0.23962	0.15307	1.000	-0.6723	0.1930
	35–44	0.04329	0.19105	1.000	-0.4967	0.5833
	45–69	0.27099	0.21970	1.000	-0.3500	0.8920
35–44	16–19	-0.55998^∗^	0.18693	0.030	-1.0883	-0.0316
	20–24	-0.28291	0.18921	1.000	-0.8177	0.2519
	25–34	-0.04329	0.19105	1.000	-0.5833	0.4967
	45–69	0.22769	0.24624	1.000	-0.4683	0.9237
45–69	16–19	-0.78767^∗^	0.21612	0.003	-1.3985	-0.1768
	20–24	-0.51061	0.21809	0.198	-1.1271	0.1058
	25–34	-0.27099	0.21970	1.000	-0.8920	0.3500
	35–44	-0.22769	0.24624	1.000	-0.9237	0.4683

*^∗^*p* < 0.05.*

**FIGURE 2:**
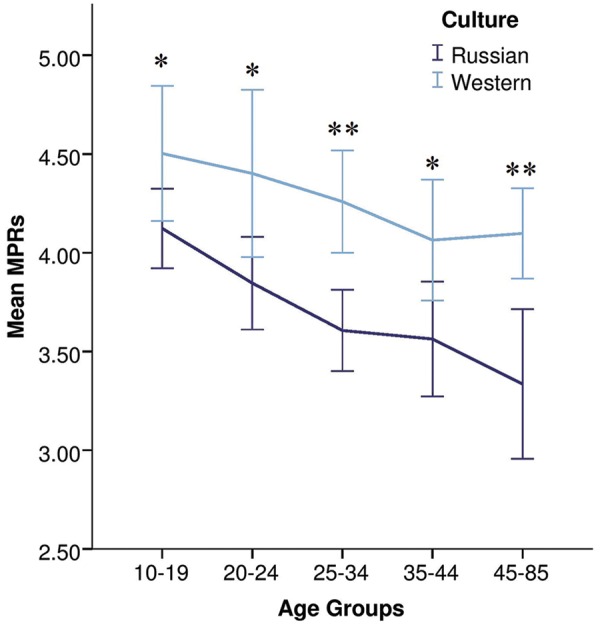
**Decreasing utilitarian judgment in older age groups in Russian and Western cultures.** Mean MPRs with 95% confidence intervals shown for five age groups: (1) 10–19, (2) 20–24, (3) 25–34, (4) 35–44, and (5) 45–85 years old. Significant age trends in Russian (Jonckheere trend test, *p* < 0.001) and Western (Jonckheere trend test, *p* = 0.007) cultures. MPRs were higher in the Western sample within all five age groups, *t*-test for independent samples, ^∗^*p* < 0.05, ^∗∗^*p* < 0.01.

As can be seen from **Figure 2**, mean MPR values decrease with age, suggesting, to some extent, that in the transition from teenager to adulthood, moral judgments become less utilitarian. This trend is observed in both cultures, Russian (Jonckheere trend test, *p* < 0.001) and Western (Jonckheere trend test, *p* = 0.007). Mean MPRs were higher in the Western sample within each of the five age groups [*t*-test for independent samples, age group 1: *t*(129) = 1.978, *p* < 0.05, *d* = 0.35; age group 2: *t*(116) = 2.402, *p* = 0.018, *d* = 0.45; age group 3: *t*(157.317) = 3.923, *p* < 0.001, *d* = 0.63; age group 4: *t*(101.661) = 2.387, *p* = 0.019, *d* = 0.47; age group 5: *t*(48.750) = 3.507, *p* < 0.001, *d* = 1.00].

**FIGURE 3:**
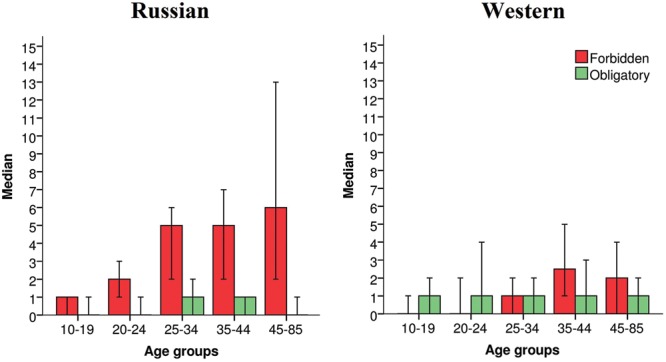
**Extreme judgments of “Forbidden” and “Obligatory” in five age groups.** Medians are shown with 95% confidence intervals for five age groups: (1) 10–19, (2) 20–24, (3) 25–34, (4) 35–44, and (5) 45–85 years old. An age trend toward increasing proportion of responses “Forbidden” is shown for both, Russian (left graph, Jonckheere-Terpstra test, *p* < 0.001) and Western (right graph, Jonckheere-Terpstra test, *p* < 0.001) samples.

The analysis of extreme judgments revealed that in both cultures, the number of “Forbidden” responses was significantly different across the five age groups (Kruskal–Wallis test, Russian sample *p* < 0.001, Western sample *p* < 0.007). However, no such difference was found in the case of “Obligatory” responses (Kruskal–Wallis test, Russian sample *p* = 0.775, Western sample *p* = 0.984). In the Russian sample, participants of all age groups, apart from the youngest (16–19 year olds), used the non-utilitarian end of the scale more often than utilitarian (Wilcoxon matched pairs test, *n*_1_ = 93, *T* = 966.5, *Z* = 0.49, *p* = 0.623; *n*_2_ = 86, *T* = 689.5, *Z* = 2.95, *p* = 0.003, *r* = 0.23; *n*_3_ = 81, *T* = 326.5, *Z* = 5.17, *p* < 0.001, *r* = 0.41; *n*_4_ = 40, *T* = 54.5, *Z* = 3.92, *p* < 0.001, *r* = 0.44; *n*_5_ = 27, *T* = 26.5, *Z* = 3.52, *p* < 0.001, *r* = 0.48). In the Western sample, the youngest age group responded “Obligatory” more often than “Forbidden”; among the other age groups, however, there was no significant difference in the two types of responses (Wilcoxon matched pairs test, *n*_1_ = 38, *T* = 98, *Z* = 2.19, *p* < 0.03; *n*_2_ = 32, *T* = 150.5, *Z* = 0.63, *p* = 0.53; *n*_3_ = 85, *T* = 843.5, *Z* = 0.313, *p* = 0.75; *n*_4_ = 109, *T* = 1263, *Z* = 0.67, *p* = 0.50; *n*_5_ = 68, *T* = 571, *Z* = 1.85, *p* = 0.06). However, there was a trend toward increasing number of “Forbidden” responses among the older age groups in both Russian (**Figure 3**, Jonckheere-Terpstra test, *p* < 0.001) and Western (**Figure 3**, Jonckheere-Terpstra test, *p* < 0.001) cultures.

### Cultural Comparisons

In general, Western subjects provided more utilitarian judgments than Russian subjects [*t*(642.451) = 4.844, *p* < 0.001, *d* = 0.38]. However, the effect of culture was gender-specific (see **Figure 1**; **Table 6**): Western men delivered more utilitarian judgments than Russian men [*t*(278) = 2.91, *p* = 0.004, *d* = 0.35], but there was no difference in women’s responses [*t*(377) = 1.66, *p* = 0.1].

**Table 6:** Descriptive statistics of MPRs for gender and culture groups.

Culture	Gender	Mean	*SE*	95% confidence interval	
				Lower bound	Upper bound
Russian	Male	3.982	0.116	3.755	4.210
	Female	3.716	0.071	3.577	3.856
Western	Male	4.443	0.079	4.288	4.598
	Female	3.889	0.092	3.708	4.070

**FIGURE 4:**
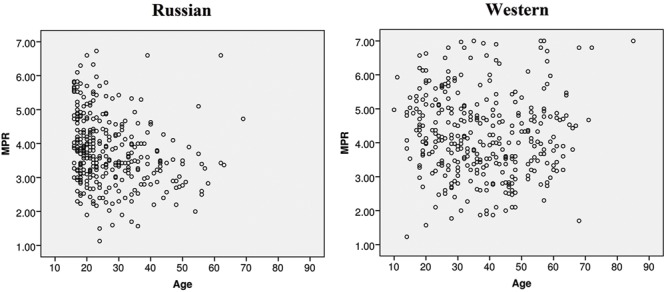
**Scatterplots showing the relationship between MPRs and age of participants in Russian and Western samples.** Western sample had a greater variance in age groups over 25 years old (Leven’s test, age group 3: *F* = 6.736, *p* = 0.01; age group 4: *F* = 5.685, *p* = 0.019; age group 5: *F* = 3.987, *p* = 0.048).

Russian and Western samples overall had different variances (**Figure 4**, Leven’s test, *F* = 10.753, *p* < 0.001) with a greater variance in Western sample. However, analyses of each age group revealed different variances within groups 3, 4, and 5 (from 25 years and older; Leven’s test, age group 3: *F* = 6.736, *p* = 0.01; age group 4: *F* = 5.685, *p* = 0.019; age group 5: *F* = 3.987, *p* = 0.048) but not within groups 1 or 2 (10–24 years old; Leven’s test, age group 1: *F* = 0.088, *p* = 0.767; age group 2: *F* = 0.316, *p* = 0.575).

Russian men, as compared to Western men, more often responded “forbidden” (Mann–Whitney *U*-test, *n*_1_ = 89, *n*_2_ = 191, *U* = 7077, *Z* = 2.33, *p* = 0.02, *r* = 0.14) and less often “obligatory” (*U* = 6626.5, *Z* = 3.05, *p* = 0.002, *r* = 0.18). Russian women were more likely to select “forbidden” than Western women (*n*_1_ = 238, *n*_2_ = 141, *U* = 13791, *Z* = 2.95, *p* = 0.004, *r* = 0.15), but no differences were observed for “obligatory” (*U* = 16753, *Z* = 0.03, *p* = 0.98).

## Discussion

The goal of this study was to explore the role of gender and age in the formation of moral judgments in two different cultures, Russian and Western. In brief, our results show both that there are principles that hold across sociocultural groups, supporting the universality thesis, and that gender, age and the East–West axis can account for some of the variance around these principles. Below, we discuss these findings and their implications for current debates about the nature of moral competence.

### Universal Moral Principles?

This study expands the cross-cultural evidence for universality of three principles of harm by showing that men and women, of different age groups, and from both Russian and Western cultures perceive that (1) means-based harms are worse than foreseen side effects, (2) actions leading to harm are worse than omissions, and (3) harm involving physical contact is worse than harm not involving contact. These results are consistent with the view that some moral principles cut across significant sociocultural variation in expressed moral judgments.

The universality claim, in its broadest sense, is the idea that certain aspects of human thought are part of our endowment, and thus species-typical. This claim does not imply that such cognitive processes are invariable in their expression. Rather, the claim is that such processes will appear in most cultures in a similar fashion, and with both predictable and constrained variability. Thus, language, music, violence, and cooperation appear in every culture, but how they are expressed varies to some extent among cultures.

Previous work, using a well-controlled battery of moral dilemmas and a web-based methodology, revealed that subjects’ moral judgments of harm are mediated by the three moral principles, irrespective of whether they are from a heterogeneous Western population (e.g., USA, UK, Canada, Australia, Dutch, urban-educated Mayan; Cushman et al., 2006; Hauser et al., 2009; Abarbanell and Hauser, 2010) or Russian population (Arutyunova et al., 2013). Similarly, Barrett et al.’ (2016) study of small scale societies shows that intentionality plays a significant role in most moral judgments, but with some variation in the magnitude of its impact. However, Barrett et al. (2016) tend to characterize the universality claim about intentionality as one in which exceptions are deflating. That is, if any exception is uncovered, the universality thesis is defeated. In contrast, our perspective on universality is that some sociocultural variance is expected, but should be constrained and predictable, once the system is well-understood. Comparing moral judgment with language, one can observe that our linguistic competence is universal and provides us with an ability to comprehend and generate linguistic expressions which are partially shaped by linguistic input. Hierarchical structure is part of all languages, but the arrangement of linguistic elements within each language may differ within limits. Thus, the seemingly universal mechanisms of moral judgment do not eliminate variation, but rather allow for a limited range of it, which we explore next.

### Sociocultural Variation of Moral Judgments

In this study we explored three sociocultural dimensions which may influence moral judgment. It has been shown that all three – gender, age and type of culture – have a significant effect on how individuals judge various harmful actions toward one individual which result in saving five other people. Particularly, we showed that in both Russian and Western cultures, men delivered more utilitarian judgments than women; and that utilitarian judgments decrease with age in Russian culture with a similar trend in Western culture. Moreover, the observed cultural variation was gender-specific with no differences in women’s responses, while Western men delivered more utilitarian judgments than Russian men. We next explore potential explanations for these patterns.

#### Gender Differences

The results of this work are consistent with prior studies showing that in hypothetical moral dilemmas involving harm, men are more likely than women to judge as permissible sacrificing one life to save many more (Fumagalli et al., 2010; Youssef et al., 2012; Friesdorf et al., 2015). In their recent work, Friesdorf et al. (2015) pointed out that they only found one study directly examining the effect of gender on moral judgments (Italian sample, Fumagalli et al., 2010). The work presented here extends this cross-cultural evidence showing that among Russians, men are more likely to provide utilitarian moral judgments than women.

A number of studies demonstrate that utilitarian decisions are associated with (1) more rational, deliberative cognitive processes and (2) reduced emotion and empathy. For example, cognitive load makes people more utilitarian and increases their response time (Greene et al., 2008); higher scores on cognitive reflection task performed prior to solving moral dilemmas correlate with utilitarian decisions (Paxton et al., 2012). At the same time, individuals with reduced empathy and impaired social emotions, such as patients with ventromedial prefrontal damage (Koenigs et al., 2007) and those suffering from trait alexithymia (Patil and Silani, 2014), are also more likely to deliver utilitarian judgments. Similarly, psychopaths, who show normal overall patterns of moral judgments (Cima et al., 2010), generate more utilitarian decisions in cases of non-personal moral dilemmas (Koenigs et al., 2012). Healthy individuals with reduced empathetic concern also deliver more utilitarian moral judgments (Gleichgerrcht and Young, 2013). Moreover, higher levels of emotional arousal measured via electrodermal activity of skin conductance are associated with a decreased likelihood of utilitarian-biased moral behavior (Navarrete et al., 2011). Enhanced emotional responses by means of serotonin administration also make people less utilitarian when they judge highly emotional personal moral dilemmas (Crockett et al., 2010).

Thus, one possible explanation for our results may be rooted in gender differences in emotion, including perhaps especially, empathy. In particular, our results suggest that men may respond with less empathy toward the individuals in the scenarios, thereby enabling a more “calculated” utilitarian judgment. Consistent with this interpretation is evidence that women report stronger emotional responses (Allen and Haccoun, 1976; Grossman and Wood, 1993; Brody and Hall, 2000; Chaplin, 2015) and greater empathetic concern (e.g., Davis, 1983; Eisenberg and Lennon, 1983; Rueckert and Naybar, 2008) than men. In a meta-analytic study applying a process dissociation method to moral judgments, Friesdorf et al. (2015) argued that a gender difference in utilitarian responses is small compared to a much stronger difference in deontological responses. The authors point out that their results correspond to the data demonstrating strong gender differences in affective processing with little gender difference in cognitive processing. Thus, according to Friesdorf et al. (2015), gender differences in moral judgments (i.e., the greater utilitarian judgment in men) may be accounted for by differences in emotional as opposed to controlled cognitive processing.

Gender differences in emotional development are considered as one of the factors underlying the formation of gender-specific behaviors (Brody, 1985). Emotions are viewed as part of socialization resulting in development of different social roles for men and women (e.g., Eagly and Wood, 1991; Grossman and Wood, 1993; Brody and Hall, 2000). “Traditionally, in Western industrial societies women are more likely than men to have domestic and nurturing roles, in which taking emotional care of others is their main task” (Fischer et al., 2004, p. 87). Moreover, “women are more likely than men to fill caretaker roles… when employed for pay (e.g., teacher and nurse)” (Grossman and Wood, 1993, p. 1010). Gilligan (1982) was the first to point out the difference in the processes of socialization of boys and girls which could cause variation in moral judgments. Although, Gilligan’s position found little support in the studies of moral development using Kohlberg’s experimental paradigm (Jaffee and Hyde, 2000), methodological differences alone may account for the differences reported here and elsewhere for weaker emphasis on utilitarianism as a guiding principle for moral judgments.

As previously mentioned, women report greater emotionality than men, especially in terms of interpersonal expression (Allen and Haccoun, 1976). As others have suggested, the interpersonal dimension is important for women who use communication to establish and enhance social connections and create relationships (Gilligan, 1982; Eagly, 1987). Men, in contrast, value their independence, aiming to achieve individual goals and pursue dominance. In studies of personality traits, women show a greater tendency to trust, are more likely to emotionally invest and affiliate with others, be respectful, and avoid taking advantage of them (Feingold, 1994; Costa et al., 2001; Weisberg et al., 2011). Moreover, these gender differences appear across different cultures, including traditional cultures, with some variation: in Western individualistic countries (Europe and USA) the magnitudes of gender differences were more pronounced (Costa et al., 2001). Women tend to need social support more than men (Tamres et al., 2002). In the context of evaluating hypothetical moral dilemmas, women are more likely to take the perspectives of more than one character while men are more likely to report taking the observer role (Pratt et al., 1987). The high importance of interpersonal relations for women is also reflected in their reporting greater empathetic concern, the ability to perceive and understand the feelings and emotions of other people (e.g., Davis, 1983; Eisenberg and Lennon, 1983; Rueckert and Naybar, 2008). Rosen et al. (2016) showed that women not only exceed men in emotional empathy but also make more altruistic moral decisions which are mediated by emotional empathy. Fumagalli et al. (2010) argued that women may be less likely to favor utilitarian outcomes than men because they are more empathic. In Harenski et al.’ (2008) fMRI study, women exhibited stronger modulatory relationships between activity in emotion and empathy-related brain areas [posterior cingulate cortex (PCC) and insula] while generating moral judgments. Men, in contrast, showed a stronger modulatory activity in inferior parietal cortex which the authors related to processing difficult contextual information.

Thus, in the case of harm-based moral dilemmas, women may perceive the interpersonal aspect of situations with greater emotions and empathy than men, which results in their rejection of harming one to save many. Men, in contrast, are more likely to see the same dilemma through the lens of quantifiable benefits, and thus tilt their judgments toward the “calculated” utilitarian outcomes.

#### Age Variation

In parallel with our comments on gender differences, one also expects variation over development even in situations where the underlying processes are universal. Decades of work on language acquisition support this position (Pinker, 1994; Yang, 2006). In the case of age, however, a significant component of the variation will be due to changes in systems outside the core competence, such as maturation of motor and sensory systems, together with gradual and slow changes in executive mechanisms (e.g., attention, working memory, self-control). Thus, age variation is expected, but the question is what kind of variation and why. The analyses presented here add to current discussions about the role of age in patterns of moral judgments.

The analysis of moral judgments across different age groups revealed similar trends within both Russian and Western cultures: the older the age group of participants, the less utilitarian judgments they expressed, and the more they used the non-utilitarian end of the scale (“forbidden”). These results suggest that as men and women mature they tend to judge utilitarian outcomes as less permissible.

Studies of adult development demonstrate an increase in emotional and cognitive reactivity to socially important issues. For example, interpersonal matters are more emotionally salient in older adulthood (Blanchard-Fields et al., 1995, 2007). Particularly, in situations involving social and personal loss and eliciting sadness, self-reported, and physiological emotional responding is higher in older adults relative to younger and middle-aged adults (Kunzmann and Gruhn, 2005; Kliegel et al., 2007; Seider et al., 2011). In general, older adults tend to prioritize positive affiliative emotions (Carstensen, 2006), for example when processing interpersonal information such as facial expressions (Mienaltowski et al., 2011). Socioemotional selectivity theory suggests that in the second half of life individual motivation is shifting from future-oriented individual goals toward social and emotional aspects of life (Carstensen et al., 2003; Carstensen, 2006). These data on enhancing reactivity within the affiliative emotional domain during lifespan may be related to a greater empathetic concern for other people. Sze et al. (2012) found that emotional empathy and prosocial behavior increase with age. Moreover age-related increases in prosocial behavior were partially accounted for by an increase of empathic concern. In line with these results, Rosen et al. (2016) showed that altruistic moral decisions also increase with age and this increase is mediated by emotional empathy. As discussed previously, enhanced emotions and empathy correlate with decreased utilitarian moral judgments.

Harenski et al. (2012) investigated patterns of brain activity while evaluating the severity of moral and non-moral transgressions in a population of participants with an age range from 13 to 53. At a behavioral level, there were no age effects. However, positive significant correlations were observed between age and activity in temporo-parietal junction (TPJ) as well as age and activity in PCC. Several studies have suggested that the TPJ is involved in theory of mind processing (e.g., Saxe and Kanwisher, 2003; Decety and Lamm, 2007), and plays a significant role in moral judgments (e.g., Young et al., 2010; Koster-Hale et al., 2013). In contrast, the PCC plays a significant role in emotional and self-reflective processing. Harenski et al. (2012) also noticed that the PCC activity increased in young adults as compared to adolescents, while TPJ activity increased later in adulthood, suggesting that the brain areas engaged in moral judgment changed during individual development and throughout life. Results of another fMRI study by Decety et al. (2012, p. 218) support the view that moral judgment requires “a complex integration between emotion and cognition that gradually changes with age.” A gradual decrease in activity of brain structures associated with emotion (amygdala and insula) in older individuals was accompanied by an increase in activity of cortical areas that have strong connections with amygdala and insula and involved in decision making (medial and ventral prefrontal cortex); these brain structures become more functionally coupled with age.

Several authors have suggested, based on both neurobiological and behavioral data, that normal lifespan development is associated with strengthening the interconnections between emotional, cognitive and behavioral domains which creates the grounds for greater empathy and more complex emotions (Magai, 2008). For example, Charles (2005) showed that emotional responding becomes more heterogeneous in older age. In situations involving injustice, older individuals often experience several different emotions at the same time while younger individuals are more likely to report a single primary emotion. Moreover, greater heterogeneity of emotions was shown to be related to a greater number of life experiences. Studies of clinical populations, including especially individuals on the autistic spectrum, reveal that lack of connectivity between key social-emotional mechanisms helps to explain the deficits (Uddin et al., 2013). In our opinion, the trend of decreasing utilitarian moral judgments with age found in our study, along with increased emotional responding and heterogeneity of emotions, socioemotional motivation, empathy, and prosocial behavior shown in other studies, may reflect developmental processes of accumulation of experience of social interactions throughout the lifespan. Several authors point out a significant increase in sociocultural experience and accumulated knowledge of the world associated with adult development (e.g., Baltes, 1987, 1993, 1997; Schaie, 1996; Kaufman and Lichtenberger, 2002). Experience in solving interpersonal problems accumulates throughout life (Heidrich and Denney, 1994) and older adults are more effective in solving interpersonal problems than younger adults (Blanchard-Fields et al., 2007). Thus, lifespan development may be associated with an increase in emotional and empathetic involvement in situations with interpersonal context, which in case of our moral dilemmas results in judging harming a person as less permissible, even when such actions bring an outcome of saving more people.

#### Cultural Differences

Looking into different cultures may provide insights into the relative plasticity of our moral judgments. In this study we showed that moral judgments of Western English-speaking individuals, in general, were more utilitarian than moral judgments of Russian individuals, i.e., Russian participants rated less permissible harming one person in order to save five others. These results correspond to the data obtained in other Eastern cultures, e.g., Chinese participants provide less utilitarian moral responses than American (Ahlenius and Tännsjö, 2012) and British (Gold et al., 2014); Korean participants when responding to moral dilemmas in their own language were shown to deliver no utilitarian choices at all (Costa et al., 2014). Therefore these results may reflect the general tendency of individuals from collectivistic cultures.

However, we also showed that such overall cultural difference was due to more utilitarian judgments of Western men as compared to Russian men while no cultural difference was observed in women. Thus, the difference in utility of moral judgment between the cultures was only true for one gender. As mentioned before, utilitarian judgment correlates with reduced emotion and empathy. Fischer et al. (2004) analyzed cross-cultural variability of gender differences in emotion across countries with different gender roles. They used the Gender Empowerment Measure (GEM) which reflects how actively women take part in economic and political life: the higher the GEM score, the more status and power women have in a particular country. The GEM score is also related to the type of culture: high GEM scores are mostly observed in Western European and English-speaking countries (see Fischer et al., 2004; United Nations Development Programme Human Development Report, 2008 for a list of GEM ratings for each county) with individualistic independent social orientation (e.g., Nisbett et al., 2001)^2^. Fischer et al. (2004) showed that respondents in countries with high GEM scores rated their powerless emotions (including affiliative emotions, such as sadness, guilt, and shame) as less intense than respondents in countries with low GEM scores. Moreover, women’s ratings of intensity of emotions were independent of their country’s GEM score and, similar to our study, this overall cross-cultural difference was due to variation in responses of men.

Restrictive emotionality in men is a typical Western phenomenon (Jansz, 2000; Fischer et al., 2004). In Western individualistic cultures, where an emphasis is made on the values of independence and autonomy (e.g., Nisbett et al., 2001), men are commonly competition-focused, a perspective that discourages powerless emotions, including affiliative emotions related to empathy (sadness, guilt, shame). Women, in contrast, are encouraged to express such emotions in order to successfully maintain social relations and fulfill their social roles. Collectivistic cultures emphasize the interdependence of individuals within a group with a special attention payed to the context of social situations, including group hierarchy (see in Nisbett et al., 2001; Fischer et al., 2004). In collectivistic countries cultural display of emotions is often similar for men and women so that “cultural norms override gender role norms” (Fischer and Manstead, 2000, p. 78). This corresponds to the previously mentioned results from Costa et al.’ (2001) study in which typical gender differences in personality traits appear across different cultures, but in Western individualistic countries these differences are more pronounced.

Fischer and Manstead (2000) found that intensity and duration of emotions was greater in collectivistic countries than in individualistic countries. These results are in line with our data on Russian men and women who were more likely to use the non-utilitarian end of the scale as compared with Western individuals. These results imply that when judging harm-based moral dilemmas, Russian individuals may experience more intense affiliative emotions and consider the situation from a more interpersonal perspective than Western individuals.

Taken together with the data on cross-cultural differences in social orientation (individualism/collectivism) and intensity of reported emotions, our results suggest that cultural differences may have a greater impact on men than on women. Supposing that women generally experience stronger affiliative emotions and are more empathetic than men (see Gender Differences), their moral responses may be less prone to cultural variation associated with the rational cognitive domain underlying deliberation, such as moral reasoning.

As shown in **Figure 4**, there was greater variation among Western than Russian respondents, but only for the older adults (over 25 years old). This cross-cultural difference can potentially be explained by different processes of cultural socialization. While Western individualistic cultures prioritize personal independent opinions, Eastern collectivistic cultures encourage individuals to decide in a manner that is best for the group and often involves compromises (e.g., Nisbett et al., 2001). This particular aspect of collectivistic cultures may account for the higher rate of conformity, the tendency to match one’s beliefs and behaviors to group norms (for a review and meta-analysis, see Bond and Smith, 1996). The lower level of variation may be the result of cultural socialization, which becomes more pronounced in adulthood. On the other hand, given that the Western sample included participants from several different countries, differences in variance could also be accounted for by the higher heterogeneity among Western respondents. We think this is less likely as the majority of respondents were from the USA, UK and Canada, which are certainly more similar than either country is to Russia.

## Conclusion

The results of the current study support the universality thesis while revealing how different factors can generate predictable patterns of variation. In particular, though Russian and Western respondents’ judgments are consistent with the three morally relevant principles developed by Cushman et al. (2006; action-omission, means-side effects and contact-non contact distinctions), gender, age, and the East–West axis directly impact the range and pattern of variance. In both, Russian and Western cultures moral judgments were more utilitarian in men than in women with a decreasing age trend. These results are consistent with the data on the role of emotion and empathy in social judgment and, in our opinion, reflect the general and gender-specific characteristics of the processes of cultural socialization during individual development.

## Author Contributions

KA drafted the work. Each of the three authors contributed to conception and design of the work; acquisition, analysis, and interpretation of data; revision of the manuscript and gave final approval of the version to be published.

## Conflict of Interest Statement

The authors declare that the research was conducted in the absence of any commercial or financial relationships that could be construed as a potential conflict of interest.

## Footnotes

1 One reason we selected MPRs as a measure was to control for a response style which has been shown to vary across cultures, with some groups (e.g., Asians) prioritizing responses around the middle of a scale in a greater degree than others (e.g., Americans), who choose extreme responses on the borders of a scale more often (see Chen et al., 1995). Our previous work on moral judgments also showed that Russian individuals overall make less extreme judgments than their Western English-speaking counterparts (Arutyunova et al., 2013). Differences in response style do not alter cross-cultural comparisons of averaged responses (Chen et al., 1995).

2 Russian Federation scored 71th in the GEM rating in United Nations Development Programme Human Development Report (2008). USA, UK, and Canada scored 15th, 14th, and 10th, respectively. Russian culture also has important features of the Eastern collectivist type of cultures (see Introduction).
